# Editorial Chit-Chat

**Published:** 1872-01

**Authors:** 


### EDITORIAL CHIT-CHAT.

DR. W. J. YOUMANS, formerly a pupil of Prof. Huxley, is the scientific editor of the *Galaxy*.

SMALL-POX.—This disease is very prevalent in many of our larger cities at present. Philadelphia seems to have suffered most from the infection. The Health Officer reported 942 cases, for the first three weeks in October last. The disease is now on the decline.

AN OLD ONE.—Solomon Bowles, Esq., has handed us a copy of the "*Ulster County Gazette*," published at Kingston, Ulster Co., N. Y., by SAMUEL FREER & SON, bearing date, Saturday, January 4th, 1800. It is in deep mourning for, and contains an account of the funeral services of, General Geo. Washington.

LOST.—In consequence of our recent fire, we have lost all copies of the BISTOURY, of number 1, volume VII. We will allow one year's subscription to a limited number who will forward us that copy. We have a few copies of all other numbers, which we can furnish to those who desire to have their volumes bound. Price, 15 cents a number.

HENRY E. DRAKE.—Have you yet called at his neat little palace of Jewelry? It's a marvel of neatness, and stocked with the rarest jems of jewelry, silver ware, watches, trinkets, and the most exquisite and delightful cloth flowers we have ever seen. Mr. Drake's taste in the selection of such articles, is proverbial. His stock is entirely new, and of the latest designs. Do not fail to call upon him in your holiday shopping excursions.

WILKIE COLLINS, is not so accurate in his scientific illustrations as was Shakespeare. The former, in his novel of "Poor Miss Finch," now being published in Harper's weekly, causes his heroine to be operated upon for congenital cataract, by a German oculist, who is made to condemn Miss Finch to six week's confinement, in a dark room, with her eye closely bandaged, before light is permitted to enter the eye. That would be considered a very great error, particularly in a German oculist, who certainly would know better. Then, the patient is permitted to recognize objects entirely too soon after the bandage *is* removed. It requires weeks and months for a patient born blind, and who has *never* seen, to become familiar with faces and forms. They have to educate their sight by the aid of touch.

Of course, this is an error that would not be discovered save by a professional man. Yet Shakespeare would never have committed a blunder of this character. His medical opinions, and scientific illustrations were faultless.

---

HOLIDAY GIFTS.—We issue our journal earlier than usual, in order to inform our patrons in this vicinity of the beautiful presents in store for them at the Jewelry establishment of HENRY W. STRANG, No. 147 Water St. Henry has a beautiful store, stocked with beautiful gifts in jewelry, chromos, flowers, vaces, watches, ornamental clocks, diamonds, jet jewelry, &c. Go see him and examine the various designs in silver and plated ware. You cannot fail to find something suitable for a gift to almost any one.

---

ELEGANT.—One of the finest stores in this country, is owned and occupied by Hall Brothers, of this city. The store room is in solid black walnut and oak, the walls beautifully frescoed, while the tables, show-cases, desks and other furniture, are both ornamental and unique. Mr. Frederic Hall has manifested great taste and talent in fitting up this palatial book store,—a place that it is a real pleasure to visit.

---

WHY NOT?—Why cannot we have a "new deal" of Lecturers in the Y. M. C. A. course, hereafter? We like Gough, Annie Dickinson, Nasby, and some others, for a dozen times or so,—but don't give them to us *every* winter. Vary the affair a little, with such names as Geo. Wm. Curtis, Olliver Wendell Holmes, Wendell Phillips, and other men of brains. Give us lecturers that can *instruct*, as well as amuse us, —but no more Gough, Dickinson and Nasby, if you please.

---

HERRING'S SAFES.—Our establishment once having been destroyed by fire, together with our books and valuable papers, we concluded to guard against a repetition of such a disaster, by securing a *fire proof* safe in which to place such articles. After examining the different safes found in the market, and learning of what their *filling* was composed, we decided upon a Herring, as the best. We ordered one of Mr. Buck, their traveling agent; it has arrived, is placed in position in our private office, where it is admired every day, for its beauty as well as its fire and burglar proof qualities. It is acknowledged to be the handsomest safe in the city, a real ornament to any gentleman's parlor. We have to thank Messrs. Herring, Farrel & Sherman, for the extra pains taken in beautifying the safe to correspond to the cosy little office in which it is placed.

---

THE INDEX.—It will be observed that we publish a complete index, with the present number of the BISTOURY, so that those of our subscribers, who have been thoughtful enough to preserve their numbers, can have them bound, when they will have a volume of 315 pages, containing information which will be sought for every day in the week. By means of the index, recipes can be instantly found, and much time and trouble saved, in looking through a number of journals for the desired information.

---

LONDON MEDICAL EXAMINATIONS.—Out of sixty-eight candidates who presented themselves before the Royal College of Surgeons, England, in May last, twenty-seven were rejected.

Judging from some of the specimens of doctors our colleges give us, those twenty-seven would have no difficulty in securing their degrees in the United States.

---

FAILURE OF CUNDERANGO.—And now the British Medical Journals are "pitching into" the cancer remedy. The British government furnished the Middlesex and St. Bartholomew Hospitals, with quantities of the bark, requesting the surgeons in charge to report upon its virtues. Their response was short, and to the point:

"Its therapeutic effects in the treatment of cancer is *nil*." But all these reports upon its worthlessness, will not prevent Dr. Bliss & Co. from making a fortune out of the sale of it.

---

ENCOURAGING—VERY.—It will be less annoying to sea side visitors, next summer, when they learn that a cheerful savant has discovered that mosquitoes are a providential guard against disease, upon the theory that in summer, the human system is peculiarly liable to fever, and he insects, by depleting the quantity of blood, get up a counter-irritation, which protects the system from attacks of noxious fevers. Of course, no one will fight the merry little creatures, after learning this.

---

FIRE PROOF DRESSES.—A Vienna Chemist has invented a composition which renders the lightest materials of stage dresses, fire-proof. An exhibition was recently given at the private theatre of Prince Lichtenstein, in which the composition was thoroughly tried. At the rise of the curtain, two life-sized dolls, dressed as ballet-dancers, were discovered. Fire was applied to both their dresses. One, was entirely consumed, while the other, to which the protective compound was applied, escaped unscorched.

---

SNAKES IN THE STOMACH.—We occasionally read highly sensational accounts in the rural papers, of some terribly afflicted woman or girl, who is the unwilling hostess of a cunning snake, which she is compelled to harbor in her stomach, and which annoys her by appearing in her mouth, nose, or ears.

Recently, one gentle Annie Brown was exhibiting her "torment" of a pet snake, which was in the habit of appearing in her mouth, much to the astonishment of the innocent country people, who were in the habit of visiting her home, in a neighboring village, for the purpose of "taking a peep" at the curious reptile. But Annie came to grief, the other day, in a very unexpected manner. A physician from Wayne county, was witnessing her performance, and when the snake protruded himself from between the girl's lips, the doctor suddenly grasped her by the throat, and choked her so severely, that the reptile could not retreat down her throat, but fell from her mouth, when it was discovered to be only an innocent india-rubber imitation of a snake, and poor Annie's occupation was gone.

---

THE WILD CHILDREN OF PITTSTON.—Since our last issue, considerable excitement has been indulged in by our Pennsylvania neighbors, over the finding of two young people, a boy and girl, 21 and 24 years of age respectively, who have been found in the woods, near Pittston, running wild. They were perfectly nude, and ran over the mountains, in search of roots, berries, &c., both winter and summer, without a stitch of clothing upon their persons. They are idiots, being unable to speak a word, and were found in a sort of rude hut, together with their father, who was engaged in boiling cracked corn, in an old kettle, while the children were shivering about the fire. They are now in the hands of a showman who is coining money by exhibiting them through the country. They are said to be great curiosities, but *should* be in a lunatic asylum, instead of an exhibition hall.

---

A TALKING MACHINE.—A Prof. Faber, of Vienna, has invented an exceedingly ingenious apparatus, which he has been exhibiting to the various Medical Colleges of the United States, during the past three months. The machine is intended to imitate the human speaking apparatus; and has a rubber mouth, the lips of which, move in imitation of human lips; the tongue is also of rubber, and the glottis is made with cords and muscles,for regulating the character of sound desired. In short,the entire machine is gotten up in imitation of the human speaking apparatus, and speaks and sings almost equal to a human being. It is worked by a lady, who makes the machine speak in the English, German, French and Italian languages. It is worked on phonetic principles, fourteen vowel and consonant words are so combined, by means of keys, which are manipulated by the operator, as to produce words and sentences. It holds conversation with its visitors, and is said to speak in a tone resembling one in the last stage of consumption. It has been recently exhibited before the classes in Bellevue Medical College, New York, and the Jefferson of Phila.

---

HOW IS THIS?—We read in the *British Medical Journal*, for July 22, 1871, a description of a "vertebrated probe and catheter," by Prof. Sayre, of New York, who claims to be the inventor of the instrument. His description is briefly as follows: "It consists simply, of a series of hollow silver disks, made a trifle smaller at one end than the other, so as to fit into one another, like a pile of cups or tumblers. These are held together by a linked chain, running through the series, and jointed nearly opposite each disk-insertion. The chain terminates in a square rod, which runs through the last disk, and is much larger than any of the others; and on the end of the small rod is cut a thread, on which runs a small button-screw, which can make the chain tight or loose, at pleasure. When the screw is turned back, the chain being lengthened, the disks fall away from one another, and the probe is as limber as a chain, capable of following any sinuosity, into which it may be pushed, or by a few turns of a screw, the disks are again drawn together, making a solid probe."

The description is illustrated by two wood cuts, showing the probe and catheter. We are a little puzzled to know who the *real* author of the "vertebrated catheter" is, as it is well known that a medical gentleman of this city claims to be the inventor of the instrument. Is it possible that Prof. SAYRE is pouching ?

---

PLUMBERS—ever watch them ?—they're funny, but his assistant or tender, more so. It's comical to see them work, provided you don't have to pay their bills. Plumber sits upon the floor, and plays with the lead pipe ;—twists, turns, hammers, punches and pounds it; then smears a lot of molten solder about a joint, with a greasy rag, which runs so smoothly and playfully, that you ache to do it yourself. Wh[illegible] this is being done, Plumber's assistant squats near by, rests his hands upon his knees, and watches the performance ; for this you pay him $3.00 per day. We have employed numerous plumbers, and paid their exorbitant bills, but for the life of us, we cannot ascertain the duties of that assistant, for we have never yet caught him doing anything but squatting upon the floor, like a kangaroo, and watching his principal. Will some one tell us what he is for, and why he persistently secures to his credit $3.00 a day, for the fun of seeing his superior work ?

